# Supplementary material for: Multiphoton imaging of neural structure and activity in Drosophila through the intact cuticle
Source: eLife. 2022 Jan 24;11:e69094. doi: 10.7554/eLife.69094 (PMC8846588; doi:10.7554/eLife.69094)
Supplement: Transparent reporting form [file elife-69094-transrepform1.docx]

***eLife’s* transparent reporting form**

We encourage authors to provide detailed information *within their submission* to facilitate the interpretation and replication of experiments. Authors can upload supporting documentation to indicate the use of appropriate reporting guidelines for health-related research (see [EQUATOR Network](http://www.equator-network.org/%20)), life science research (see the [BioSharing Information Resource](https://biosharing.org/)), or the [ARRIVE guidelines](http://www.plosbiology.org/article/info:doi/10.1371/journal.pbio.1000412) for reporting work involving animal research. Where applicable, authors should refer to any relevant reporting standards documents in this form.

If you have any questions, please consult our Journal Policies and/or contact us: [editorial@elifesciences.org](mailto:editorial@elifesciences.org).

**Sample-size estimation**

- You should state whether an appropriate sample size was computed when the study was being designed
- You should state the statistical method of sample size computation and any required assumptions
- If no explicit power analysis was used, you should describe how you decided what sample (replicate) size (number) to use

Please outline where this information can be found within the submission (e.g., sections or figure legends), or explain why this information doesn’t apply to your submission:

Sample sizes are estimated based on previous papers published and the literature.

Cuticle transmission data: Sample sizes and measurement numbers are stated in the legend of Figure 1, Figure 1-Source Data and in the methods section.

Whole brain depth analysis data: Sample sizes and attenuation measurements are stated in the legend of Figure 3, Figure 3-Source Data and in the methods section.

Simultaneous 2P and 3P functional imaging: Sample sizes and stimulation numbers are stated in the legend of Figure 5, Figure 5-Source Data and in the methods section.

2P functional imaging in behaving flies: Sample sizes and stimulation numbers are stated in the legend of Figure 6, Figure 6-Source Data1-2 and in the methods section.

Long term 2P imaging: Sample sizes and stimulation numbers are stated in the legend of Figure 7, Figure 7-Source Data 1-5 and in the methods section.

**Replicates**

- You should report how often each experiment was performed
- You should include a definition of biological versus technical replication
- The data obtained should be provided and sufficient information should be provided to indicate the number of independent biological and/or technical replicates
- If you encountered any outliers, you should describe how these were handled
- Criteria for exclusion/inclusion of data should be clearly stated
- High-throughput sequence data should be uploaded before submission, with a private link for reviewers provided (these are available from both GEO and ArrayExpress)

Please outline where this information can be found within the submission (e.g., sections or figure legends), or explain why this information doesn’t apply to your submission:

Biological replicates are measurements or recordings on distinct biological preparations or animals.

Technical replicates are measurements or stimulations done within one animal.

The data reported in this study consists of biological replicates and technical replicates.

For functional imaging experiments at least n=3 animals were used. Each animal was stimulated 3-5 times with the same stimulus. Details are found in the methods section.

For cuticle transmission experiments, n=4-5 animals were used. Each cuticle sample was used for multiple measurements in different locations of the cuticle. Details are found in the methods section.

**Statistical reporting**

- Statistical analysis methods should be described and justified
- Raw data should be presented in figures whenever informative to do so (typically when N per group is less than 10)
- For each experiment, you should identify the statistical tests used, exact values of N, definitions of center, methods of multiple test correction, and dispersion and precision measures (e.g., mean, median, SD, SEM, confidence intervals; and, for the major substantive results, a measure of effect size (e.g., Pearson's r, Cohen's d)
- Report exact p-values wherever possible alongside the summary statistics and 95% confidence intervals. These should be reported for all key questions and not only when the p-value is less than 0.05.

Please outline where this information can be found within the submission (e.g., sections or figure legends), or explain why this information doesn’t apply to your submission:

Comparisons with one variable were first analysed using one-way ANOVA followed by Tukey’s multiple comparisons post-hoc test.

Comparisons with more than one variable were first analysed using two-way ANOVA followed by Tukey’s multiple comparisons post-hoc test.

Paired t-test was used for comparing fly’s locomotion behaviors within a trial.

Statistical tests used in each Figure are stated in each legend and the details are explained in the methods section and p- values are reported in respective source data files.

(For large datasets, or papers with a very large number of statistical tests, you may upload a single table file with tests, Ns, etc., with reference to sections in the manuscript.)

**Group allocation**

- Indicate how samples were allocated into experimental groups (in the case of clinical studies, please specify allocation to treatment method); if randomization was used, please also state if restricted randomization was applied
- Indicate if masking was used during group allocation, data collection and/or data analysis

Please outline where this information can be found within the submission (e.g., sections or figure legends), or explain why this information doesn’t apply to your submission:

For all of the experiments performed in the manuscript, subjects used in the experiments were selected randomly from the indicated genotype of flies.

**Additional data files (“source data”)**

- We encourage you to upload relevant additional data files, such as numerical data that are represented as a graph in a figure, or as a summary table
- Where provided, these should be in the most useful format, and they can be uploaded as “Source data” files linked to a main figure or table
- Include model definition files including the full list of parameters used
- Include code used for data analysis (e.g., R, MatLab)
- Avoid stating that data files are “available upon request”

Please indicate the figures or tables for which source data files have been provided:

**Figure_1_source_data_1**: Source data for plots Figure 1D and 1G.

**Figure_3_source_data_1**: Source data for plots Figure 3B and 3C.

**Figure_3-figure_S1_source_data_1:** Source data for plots for Figure 3-Supp 1C, D.

**Figure_3-figure_S2_source_data_1:** Source data for plots for Figure 3-Supp 2B-H.

**Figure_3-figure_S3_source_data_1:** Source data for plots for Figure 3-Supp 3B-H.

**Figure_3-figure_S4_source_data_:** Source data for plots for Figure 4-Supp 4C, D.

**Figure_4_source_data_1:** Source data for plots for Figure 4A-D.

**Figure_5_source_data_1:** Source data for plots Figure 5G-5I.

**Figure_6**_**source_data_1**: Source data for plots Figure 6B and 6C.

**Figure_6**_**source_data_2**: Source data for plots Figure 6E-6K.

**Figure_7**_**source_data_1_1-4**: Source data for plots Figure 7B-7E.

**Figure_7**_**source_data_1_5**: Source data for plot Figure 7F.

Matlab code for functional imaging analysis.
